# Therapeutic efficacy of intra-articular hyaluronan derivative and platelet-rich plasma in mice following axial tibial loading

**DOI:** 10.1371/journal.pone.0175682

**Published:** 2017-04-13

**Authors:** Xin Duan, Linda J. Sandell, Nobuaki Chinzei, Nilsson Holguin, Matthew J. Silva, Antonella Schiavinato, Muhammad Farooq Rai

**Affiliations:** 1 Department of Orthopaedic Surgery, Musculoskeletal Research Center, Washington University School of Medicine at Barnes-Jewish Hospital, St. Louis, Missouri, United States of America; 2 Department of Orthopedic Surgery, First Affiliated Hospital of Sun Yet-san University, Guangzhou, China; 3 Department of Cell Biology and Physiology, Washington University School of Medicine at Barnes-Jewish Hospital, St. Louis, Missouri, United States of America; 4 Department of Biomedical Engineering, Washington University in St. Louis, St. Louis, Missouri, United States of America; 5 Fidia Farmaceutici S.p.A., Abano Terme, Padova, Italy; University of Rochester, UNITED STATES

## Abstract

**Objective:**

To investigate the therapeutic potential of intra-articular hyaluronan-derivative HYADD^®^ 4-G and/or platelet-rich plasma (PRP) in a mouse model of non-invasive joint injury.

**Methods:**

Non-invasive axial tibial loading was used to induce joint injury in 10-week-old C57BL/6J mice (n = 86). Mice underwent a single loading of either 6 Newton (N) or 9N axial tibial compression. HYADD^®^ 4-G was injected intra-articularly at 8 mg/mL or 15 mg/mL either before or after loading with or without PRP. Phosphate-buffered-saline was injected as control. Knee joints were harvested at 5 or 56 days post-loading and prepared for micro-computed tomography scanning and subsequently processed for histology. Immunostaining was performed for aggrecan to monitor its distribution, for CD44 to monitor chondrocyte reactive changes and for COMP (cartilage oligomeric matrix protein) as an index for cartilage matrix changes related to loading and cartilage injury. TUNEL assay was performed to identify chondrocyte apoptosis.

**Results:**

Loading initiated cartilage proteoglycan loss and chondrocyte apoptosis within 5 days with slowly progressive post-traumatic osteoarthritis (no cartilage degeneration, but increased synovitis and ectopic calcification after 9N loading) at 56 days. Mice treated with repeated HYADD^®^ 4-G (15 mg/mL) or HYADD^®^ 4-G (8 mg/mL) ± PRP or PRP alone exhibited no significant improvement in the short-term (5 days) and long-term (56 days) consequences of joint loading except for a trend for improved bone changes compared to non-loaded joints.

**Conclusion:**

While we failed to show an overall effect of intra-articular delivery of hyaluronan-derivative and/or PRP in reversing/protecting the pathological events in cartilage and synovium following joint injury, some bone alterations were relatively less severe with hyaluronan-derivative at higher concentration or in association with PRP.

## Introduction

Osteoarthritis (OA) is a leading cause of disability worldwide. Post-traumatic OA (PTOA) in particular is a frequent cause of disability following trauma to the weight-bearing joints. It is estimated that 12% of the nearly 21 million Americans with symptomatic OA have a post-traumatic etiology [1]. Joint trauma can lead to a spectrum of acute lesions, including cartilage degradation, bone remodeling, ligament or meniscus tears, and synovitis, all of which are potentially associated with PTOA.

Murine models of knee injury are critical tools to study alterations associated with PTOA in the joint. Several invasive injury models such as injecting collagenase into the joint [2], using a needle to induce anterior cruciate ligament (ACL) transection in the closed knee, or using surgical techniques to transect or disrupt the ligaments or the medial meniscus of the knee [3, 4] have been widely used. However, these models do not mimic clinically relevant injury conditions due to invasive nature and non-physiologic injury methods. Therefore, the non-invasive model described in this paper and used by others [5, 6] and us in a modified form is especially attractive for studies of early and late pathological events following knee joint injury. However, the mechanisms, by which severity of injury leads to increased cartilage degeneration and accelerated synovial response, remain incompletely understood. In addition, detection of the early events in chondrocyte responses that lead to cartilage degeneration and eventually to development of PTOA has also been difficult to accomplish. With limited treatment options and no disease-modifying OA drugs, there is a critical need to develop new therapeutic approaches to repair joint injury and reduce the probability of PTOA. The wide spectrum of changes in the knee joint following tibial loading may be targets for treatment options.

Hyaluronan (hyaluronic acid, HA) is a high molecular-weight, non-sulfated, unbranched glycosaminoglycan [7] that plays a significant role in maintaining the homeostasis of the synovial joint as well as providing lubrication, shock absorption, elasticity and hydration to the joint tissues [8], and has well known anti-inflammatory [9] and anti-apoptotic effects on chondrocytes [10]. Numerous experimental and clinical studies suggest that intra-articular injection of HA and its derivatives potentially exert a protective effect on joint structure and function [11–13]. HYADD^®^ 4-G (Fidia Farmaceutici, Abano Terme, Italy) hydrogel is a novel HA amide derivative in which an aliphatic amine is bound to HA at the carboxylic group of the glucuronic acid in only 2% of the carboxylic groups, therefore 98% remains unmodified HA. This small chemical modification on the molecule increases the viscoelastic properties of HA and thereby increasing beneficial effects in human OA as well as in surgical models of PTOA in large animals [12, 14]. Increasing the viscoelastic properties of HA provides superior lubricant properties and thus reduces the coefficient of friction as well as possibly limits articular cartilage damage during joint activity [15].

Platelet-rich plasma (PRP) is another option for the intra-articular treatment of OA, considering the high content of growth factors in the PRP preparations. The growth factors, stored in the granules of the platelets are released after platelet activation, which can occur before injection by adding thrombin or CaCl_2_. Most of these factors are implicated in the regulation of articular chondrocyte anabolic activity. In particular, platelet-derived growth factor (PDGF) stimulates chondrocyte proliferation and proteoglycan synthesis (reviewed in [16]) and transforming growth factor beta 1 (TGF-β1) affects chondrocyte proliferation [17] and stimulates chondrogenesis of stem cells.

A systematic review of the efficacy of PRP has concluded that multiple sequential intra-articular PRP injections might have beneficial effects in the treatment of mild to moderate knee OA in adult patients [18] in comparison to HA or saline. However, in animal models while the HA efficacy on experimental OA is widely reported [19, 20] data on the PRP effect are scarce [21].

To date, there is no study that has tested the potential (therapeutic) effects of HA or PRP in the injured mouse knee joint after mechanical loading. Here we have analyzed joint tissues to determine whether treatment with HA or growth factor-rich preparations administered at the time of injury or later could reduce the immediate effects of loading and/or the long-term effects of joint injury.

## Materials and methods

### Study design

**Fig 1:**
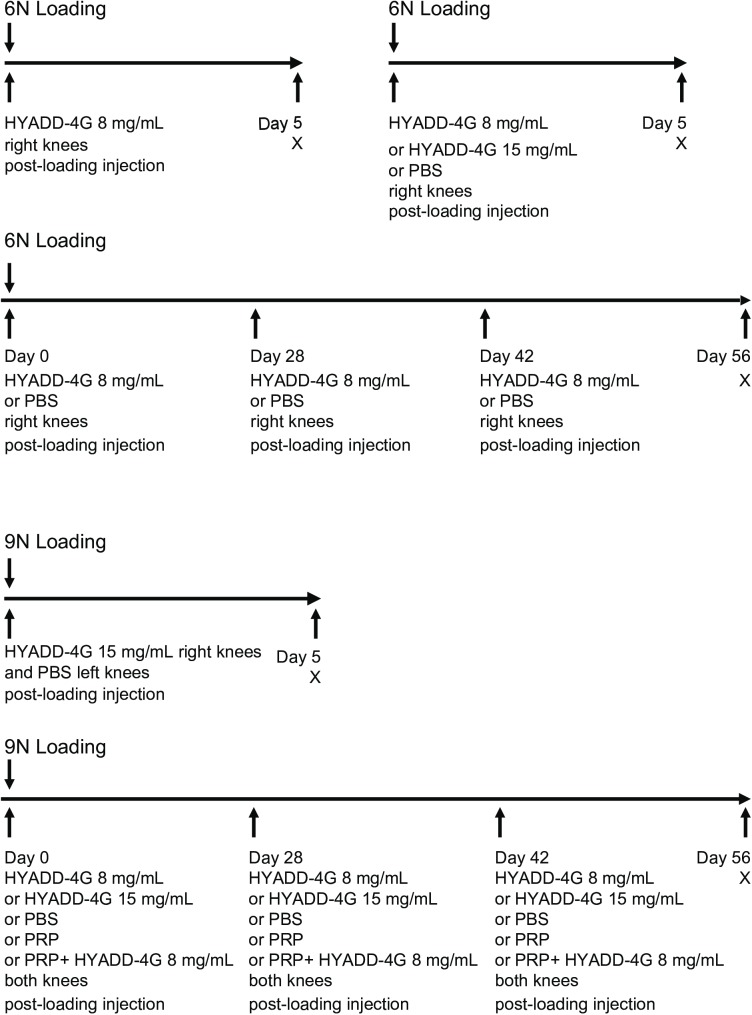
Study design. An overview of treatment(s) before and/or after loading, time points of repeated injections and analysis of 6N and 9N loading is shown. X = sacrifice, PBS = phosphate buffered saline, PRP = platelet-rich plasma.

Study procedures were approved by Washington University Institutional Animal Care and Use Committee. Mice (C57BL/6J) were procured from the Jackson Laboratory (Bar Harbor, ME) and housed under standard sanitary and husbandry conditions as described previously [22]. Briefly, mice were housed in a designated mouse facility operating at controlled temperature (21°C-22°C) and controlled lighting (12 h light, 12 h dark) conditions with high standards of sanitation. On an average 4–5 mice were kept in individually ventilated cages. Mice were provided standard rodent chow (Purina 5053, Purina Mills St. Louis, MO) and water *ad libitum*. In total, we evaluated 150 knees from 86 mice (5–14 knees per group). Some of the knees were excluded from the analysis due to the lack of an ACL rupture (in 9N loading group, in 6N loading group no ACL tear occurred) or due to lack of extraction of reliable data–for example poor tissue preparation, problems with embedding, poor orientation of sections and improper scanning. The number of mice in each group, time points and treatment options are depicted in Table 1, and an overview of the experimental design is shown in Fig 1.

**Table 1:** Loading regimens, treatment options, time points and sample size.

Force	Description of treatment	Volume injected^#^	Time point (day)	Mouse (*n*)	Knee (*n*)
	**Right knees loaded and injected only**				
6N	HYADD-4G 8 mg/mL pre-loading	15 μL	5	5	5
6N	HYADD-4G 8 mg/mL post-loading	15 μL	5	6	6
6N	PBS, control	15 μL	5	6	6^a^
6N	HYADD-4G 15 mg/mL post-loading	15 μL	5	5	5
6N	PBS control	15 μL	56	6	6^b^
6N	HYADD-4G 8 mg/mL post-loading	15 μL	56	6	6
	**Both knees loaded and injected**				
9N	HYADD-4G 15 mg/mL (right knees) and PBS (left knees) post-loading	15 μL	5	10	6 (Right)^c^, 7 (left)^d^
	**Both knee loaded and injected**				
9N	HYADD-4G 8 mg/mL, 3 injections post-loading	15 μL	56	10	14^e^
9N	HYADD-4G 15 mg/mL, 3 injections post-loading	15 μL	56	8	15*
9N	PBS control, 3 injections post-loading	15 μL	56	8	14*
9N	PRP, 3 injections post-loading	15 μL	56	8	13*
9N	PRP + HYADD-4G 8 mg/mL, 3 injections post-loading	5 μL + 10 μL mixture	56	8	13*

a: 6 left knees from this group were used for non-loaded control at day 5

b: 6 left knees from this group were used for non-loaded control at day 56

c: 3 knees were excluded due to lack of micro-CT data, 1 knee without ACL rupture was also excluded.

d: 1 knee was excluded due to lack of micro-CT data, 2 knees without ACL rupture were also excluded.

e: 2 knees were excluded due to lack of micro-CT data, 4 knees without ACL rupture were also excluded.

*: all the knees without ACL rupture were excluded

#: volume that was injected into each knee

### HYADD^®^ 4-G (HA-derivative)

HA-derived HYADD^®^ 4-G hydrogel is a non-cross-linked HA (500–730 kDa) of fermentative origin. Fidia Farmaceutici (Abano Terme, Italy) provided HA-derived HYADD^®^ 4-G hydrogel in sealed syringes with two different concentrations: 8 mg/mL and 15 mg/mL. For the sake of simplicity, we used the term HA-derivative for this product. HYADD^®^ 4-G (8 mg/mL) is commercially available with the brand name HYMOVIS^®^ (http://hymovis.com).

### Preparation of PRP

We prepared PRP from mouse blood for therapeutic use alone or combined with the HA-derivative. PRP was prepared as described previously [23]. Briefly, intra-cardiac whole blood sample was drawn from 8-week old C57BL/6J mice (n = 10) using sterile syringe pre-loaded with heparin. First, the blood was centrifuged at 200g for 20 min to separate the platelet-poor plasma from the red and white blood cells. In second step, the plasma was centrifuged at 2000g for 10 min. Platelets were re-suspended in supernatant to obtain PRP (about 10% volume of the pooled whole blood). The concentrations of the growth factors were assessed by enzyme-linked immunosorbent assay using 3 replicates. The PRP preparation had higher concentrations (mean and 95% confidence interval, CI) of growth factors namely TGF-β1 (46.87 ng/ml, 95% CI, 39.97–53.77) and PDGF AB (2.68 ng/ml, 95% CI, 2.67–2.69) compared to platelet-poor plasma, which had significantly lower concentrations (4.39 ng/ml, 95% CI, 4.24–4.54 and 0.04 ng/ml, 95% CI, 0.01–0.07 respectively).

### Axial tibial loading and intra-articular injection

Mouse knees underwent 6N or 9N of compressive loading as detailed previously [24, 25]. Briefly, under anesthesia (2.5% isoflurane in 4 L/min oxygen; Highland Medical Equipment), the right tibia was held vertically in an upside-down position between the two custom-made cups of a materials testing machine (Instron ElectroPulse E1000). Axial tibial load was applied through the ankle. Cyclic loads consisting of 0.34 sec of rise and fall time and a baseline hold time of 10 sec between each cycle for 60 cycles. Peak loading force (6N or 9N) with a 0.5N preload force (to maintain the tibia in position) was applied. Some contralateral non-loaded knees served as control.

While mice were under anesthesia, they were injected with the indicated treatment(s) (Table 1). Intra-articular injection was performed as reported by Diekman et al., [26]. Briefly, the mouse was positioned for lateral entry of needle with the left hind limb extended to facilitate injection of 15 μL into the joint space through the patellar tendon. This delivery technique does not result in initiation of OA [27]. As stated above, the volume of each treatment injected was 15 μL, however, when we combined the treatments, we mixed 5 μL of PRP + 10 μL of HYADD-4G 8 mg/mL.

### Knee joint preparation

Mice were sacrificed using CO_2_ chamber at indicated time points (Fig 1). Knee joints were dissected and fixed in 10% neutral buffered formalin. Joints were then washed with distilled water and incubated in 70% ethanol until scanning for micro-CT or decalcification. Based on our and others studies [5, 25], all of the following parameters were studied in femur.

### Micro-CT analysis

Prior to decalcification, knees were scanned using vivaCT-40 micro-CT scanner (Scanco-Medical) for analysis of 3-dimensional bone structure for trabecular bone volume fraction (BV/TV) [22, 28]. Subchondral bone plate thickness was measured via custom-written MATLAB-2015b (Mathworks) program [29].

### Histology and scoring of proteoglycan loss

Knee joints were decalcified using Immunocal (StatLab, McKinney, TX) and paraffin embedded. Five-micron thick sections were cut in sagittal plane throughout the joint with every third section stained with Safranin-O. As there was no appreciable structural loss of non-calcified cartilage, we scored articular cartilage for proteoglycan depletion (loss) using a supplementary OARSI (Osteoarthritis Research Society International) scoring system (called proteoglycan scoring system) as described by Glasson and co-workers [30]. Synovial pathology was measured as before [25, 31] using three selected sections from each knee.

### Detection of chondrocyte apoptosis

Chondrocyte apoptosis was detected by in situ terminal deoxynucleotidyltransferase-mediated dUTP nick-end labeling (TUNEL) assay (In situ Cell Death Detection Kit, Roche, Indianapolis, IN). In order to compare the number of apoptotic cells, we examined the numbers of TUNEL-positive cells in non-calcified cartilage as it is commonly thought that apoptosis naturally occurs in calcified cartilage [32]. Images acquired from three TUNEL-stained slides were evaluated by two blinded observers as described previously [33].

### Immunostaining of extracellular matrix proteins and CD44

To assess the expression pattern of extracellular matrix proteins such as aggrecan, and cartilage oligomeric matrix protein (COMP), and CD44 (a cell surface antigen for hyaluronic acid binding), we used antibody-specific immunostaining as described elsewhere [24, 25]. Selected slides were set for baking at 60°C for 4 h followed by deparaffinization in xylene twice for 5 min. Slides were then rehydrated in descending concentrations of ethanol with final washes with distilled water and phosphate buffered saline (PBS). For antigen retrieval, the slides were digested with 10 μg/mL proteinase K at 37°C for 20 min in a customized humidifying chamber. After washing with PBS, slides were blocked in 10% normal goat serum in PBS at room temperature for 1 h. After draining off the blocking buffer, slides were incubated with following primary antibodies (procured from Abcam, Cambridge, MA unless indicated otherwise) for respective proteins at 1:100 dilutions using 1.5% goat serum in PBS: rabbit anti COMP (Kamiya Biomedical Co., Seattle, WA, PC-140), rabbit anti aggrecan for G2 domain (generous gift from Dr. Amanda Fosang, Melbourne, Australia), rabbit anti CD44. Slides were incubated at 4°C overnight followed by 3 washes with PBS. The secondary antibodies were used (procured from Abcam, Cambridge, MA) at 1:200 dilutions using 2.5% goat serum in PBS. Slides were incubated at room temperature for 60 min. Finally, the slides were rinsed with PBS and distilled water before being sealed with VECTASHIELD antifade mounting medium with DAPI (Vector Laboratories, Burlingame, CA). Sections were imaged using Eclipse E800 microscope (Nikon, Tokyo, Japan) with QImaging Retiga 2000R Fast 1394 camera and MetaMorph software v7.7 (Molecular Devices, Sunnyvale, CA). All antibodies used in this study have been reported by Duan et al. [25].

### Statistical analysis

Data collection and analysis were performed in a blinded fashion. As noted above (Table 1), we used a sample size of 5–14 per group. Post-hoc power analysis showed that this sample size is sufficient to detect an effect size ranging between 1.1 to 2.0 at a power of 80% and α = 0.05 for any given parameter. Therefore, our interpretation of results and conclusion drawn are based on this level of effect size. We used one-way analysis of variance with Tukey’s honestly significant difference (HSD) post-hoc test to compare each group to all other groups in a particular parameter using GraphPad Prism v.5.04 (GraphPad Software Inc., La Jolla, CA). Data are expressed as mean ± 95% CI. Differences were considered statistically significant at P < 0.05.

## Results

### Effect of treatments on cartilage injury and proteoglycan loss

**Fig 2:**
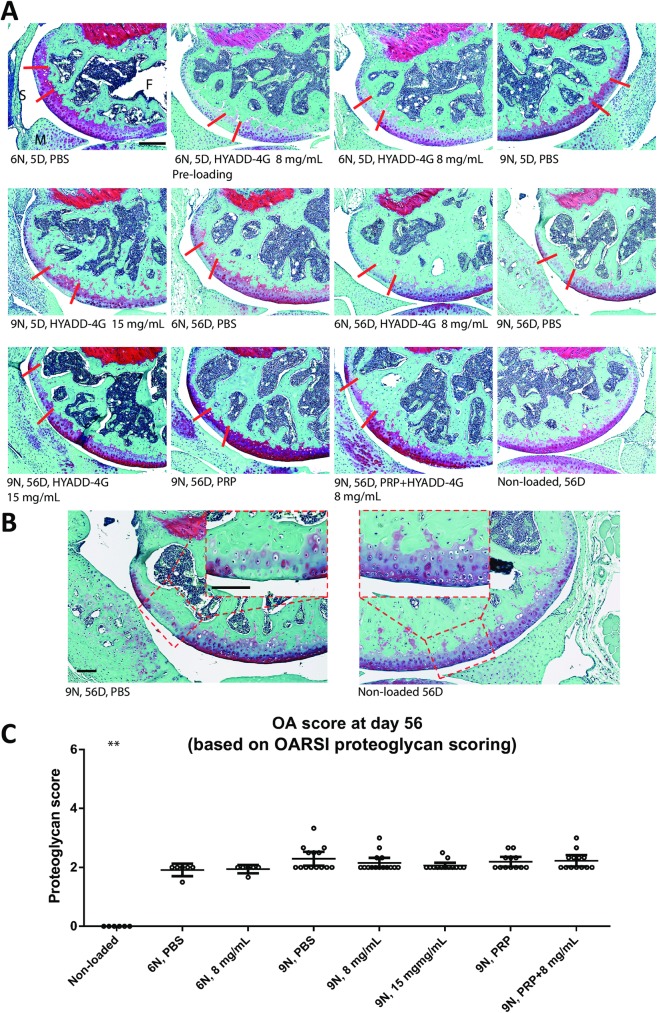
Cartilage injury. Representative images of cartilage injury of the lateral femoral condyle in each group showing loss of Safranin-O staining and absence of normal nuclear staining are provided for higher (A) and lower (B) magnification. There was no therapeutic effect of HYADD^®^ 4-G or PRP on cartilage injury and results were not different from PBS treatment. The area between two red lines in each image shows the injury site of each knee. OA scored based on proteoglycan loss on day 56 after loading showed that there was no significant effect of any treatment modality on proteoglycan replacement (C). Bar = 50 μm, ** indicates P < 0.01 compared with other groups. Error bars represent 95% confidence interval. PBS = phosphate buffered saline, PRP = platelet-rich plasma, F = femur, M = meniscus, S = synovium.

The cartilage injury site was located at the posterior aspect of the lateral femoral condyle and was characterized by the loss of Safranin-O staining without disrupting nuclear structure. We observed complete loss of Safranin-O staining indicative of proteoglycan depletion in the non-calcified cartilage extending up to 50% of the articular surface (Fig 2A and 2B). The cartilage injury was identical in 6N and 9N loading groups. We found that treatment with HA-derivative or PRP alone or in combination did not confer any protective response on cartilage proteoglycan loss (Fig 2A and 2B). We also failed to appreciate any significant differences in proteoglycan loss (at 56-days) between any treatment modalities used or between 6N and 9N loading groups (Fig 2C).

### Effect of treatments on COMP and apoptosis

**Fig 3:**
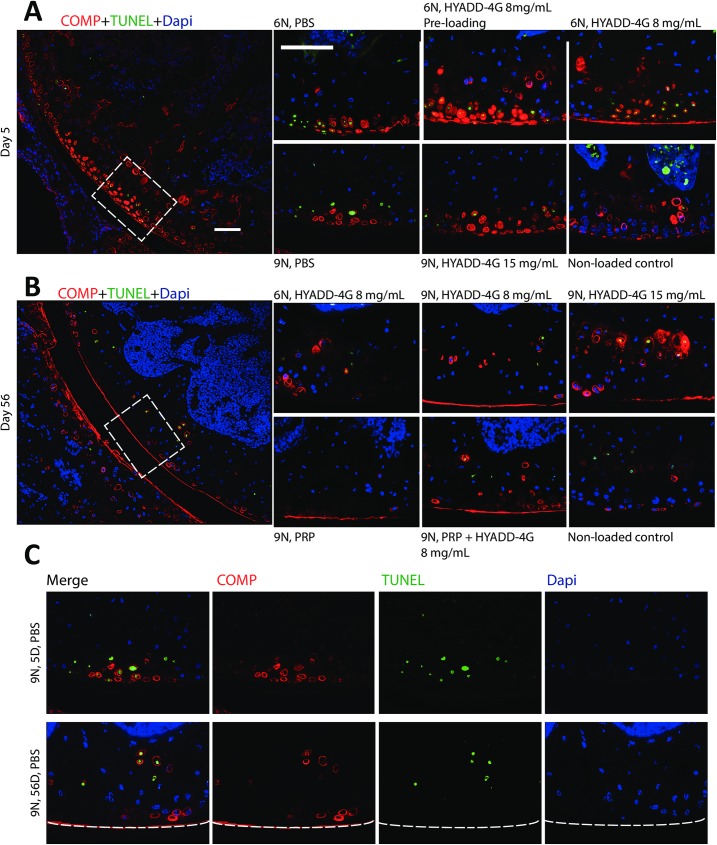
Chondrocyte apoptosis and COMP staining. Representative images of chondrocyte apoptosis (green), COMP (red) and DAPI (blue) immunostaining are shown. At day 5 (A), TUNEL assay identified apoptotic chondrocytes (green) in the injured cartilage area in all loaded groups. COMP (red) was found around the chondrocytes in the intact area and in the lacuna left by the apoptotic chondrocytes in the injured area. At day 56 (B), TUNEL positive chondrocytes in the non-calcified layer of injured cartilage disappeared. No new developed apoptosis was observed. COMP (red) was hardly seen in the injured region and its expression remained the same as day 5 in the adjacent healthy cartilage surface. None of the treatments conferred any protective response on chondrocyte apoptosis or the expression pattern of COMP at day 5 (A) and 56 (B) after loading. White boxes indicate the injured area where enlarged images were taken. Representative images at days 5 and 56 for 9N loading with split channels to show the changes of chondrocyte apoptosis (TUNEL positive cells) and distribution of COMP (red) are also presented (C). Bar = 100 μm, dotted white lines indicate the joint surface. COMP = cartilage oligomeric matrix protein, PBS = phosphate buffered saline, PRP = platelet-rich plasma, TUNEL = terminal deoxynucleotidyltransferase-mediated dUTP nick-end labeling.

Non-immune controls are shown in the S1A and S1B Fig. Immunostaining of COMP (Fig 3A–3C, red) showed its presence around the chondrocytes in the intact area and in the lacuna left by the apoptotic chondrocytes in the injured area at the early time point (day 5) post-injury. This distribution pattern of COMP is parallel with our previous findings [24, 25]. COMP staining became weaker over time and was hardly seen in the injured area at day 56 post-loading. We also observed that cells at the site of injury underwent apoptosis as TUNEL assay detected fragments of damaged DNA (Fig 3A–3C, green). There was no apoptosis in the non-calcified layer of the cartilage in the non-loaded knees and in the non-injured cartilage of the loaded knees. Apoptotic chondrocytes were randomly seen in the calcified layer of the cartilage of both loaded and non-loaded knees across all the time points. There were more apoptotic chondrocytes detected at day 5 in the non-calcified layer of the injured cartilage, which were largely cleared by day 14 (not shown) and absent at day 56. HA-derivative or PRP alone or in combination did not protect or reverse chondrocyte apoptosis.

### Effect of treatments on aggrecan and CD44

**Fig 4:**
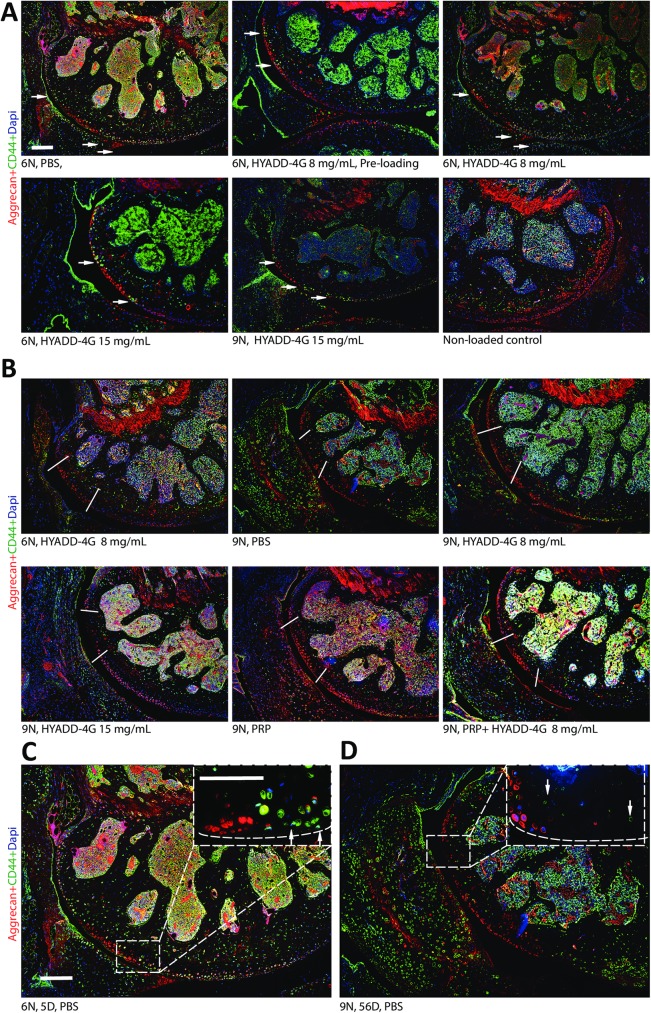
CD44 and aggrecan staining. At day 5, CD44 (green) was mainly seen in the calcified zone of articular cartilage in control knees with only a few CD44-positive signals around hypertrophic chondrocytes (A). CD44 signals were not found at the site of cartilage injury, albeit higher signals were discovered in the chondrocytes immediately around the injured zone (white arrows). There was no difference in the pattern of CD44 expression between different treatment groups. Aggrecan staining (red) was found around the chondrocytes in the intact area and in the lacuna left by the apoptotic chondrocytes in the injured area. Bar = 100 μm. At day 56, CD44 in the loaded knees, same as non-loaded control knees, was mainly seen in the calcified zone of articular cartilage with only a few CD44-positive signals around hypertrophic chondrocytes. No substantial CD44 signals were found in the chondrocytes immediately around the injured zone as day 5. There was no difference in the pattern of CD44 expression between different treatment groups. Aggrecan staining (red) was much weaker in the injured region but remain the same in the intact area of the loaded cartilage. White bars indicate the injured areas (B). Representative images of day 5 (C) and day 56 (D) of injured knees. White dotted boxes indicate the enlarged area. The white arrows in C indicate the higher CD44 signals in the chondrocytes immediately around the injured zone at day 5 while the white arrows in D show the expression of CD44 in the hypertrophic chondrocytes in both loaded and non-loaded knees at day 56. Also, low intensity of aggrecan signal in the injured region at day 56 was noticed. Dotted lines indicate the joint surface. PBS = phosphate buffered saline, PRP = platelet-rich plasma.

As reported by us [24, 25] and as our current data suggest, aggrecan, similar to COMP, was expressed around the chondrocytes in the intact cartilage area in loaded and non-loaded knees. After injury, increased aggrecan was observed in the lacunae created by apoptotic chondrocytes in the injured cartilage (Fig 4A–4C and S2A Fig, red). Furthermore, CD44, which is a cell surface receptor for HA, demonstrated a unique expression pattern (Fig 4A–4C and S1A Fig, green). Its fluorescent intensity increased in the area closely surrounding the injured cartilage at day 5 in all treatment groups (Fig 4A–4C arrows). However, this expression pattern of CD44 disappeared at day 56 (Fig 4B). CD44 chondrocytes were mostly seen in the hypertrophic cells in the calcified cartilage in all groups. As stated above for COMP and apoptosis, none of the treatments modified the expression pattern of aggrecan and CD44.

### Effect of treatments on synovitis and ectopic calcification

**Fig 5:**
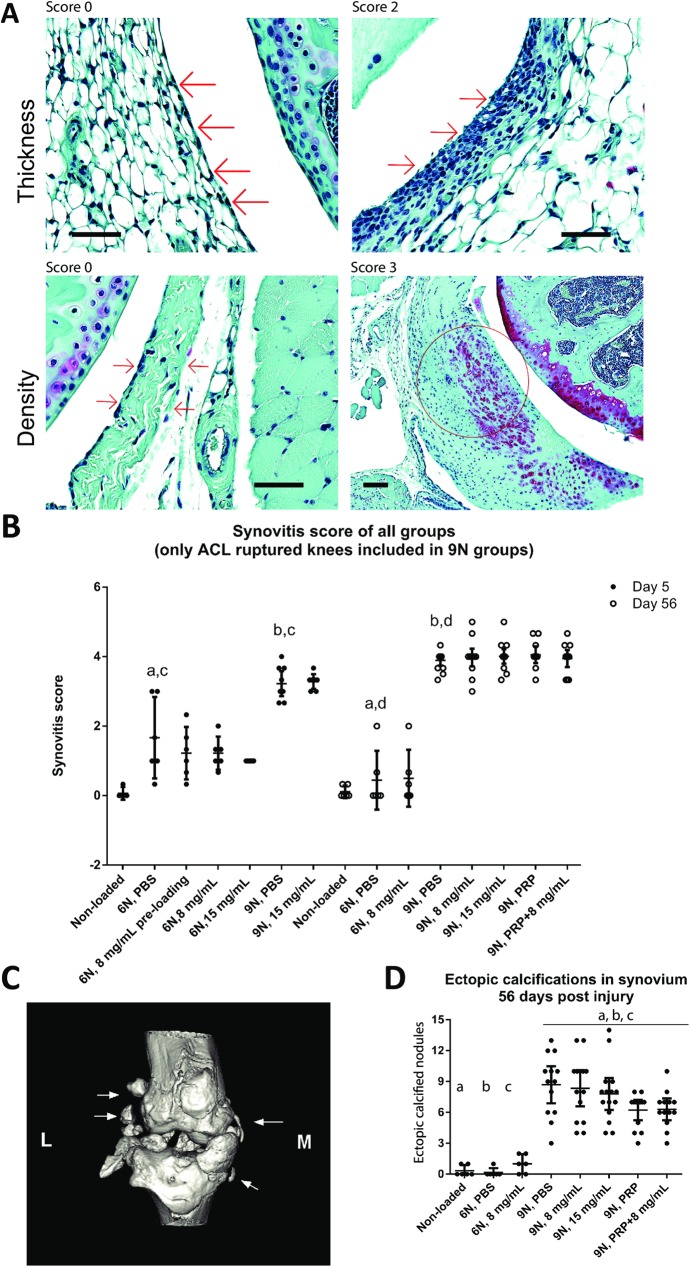
Synovitis and ectopic ossification. Enlargement of the synovial lining cell layer and density of the cells in the synovial stroma (red arrows) were individually assessed. The red circle shows a representative ectopic ossification nodule (A). Quantification of synovitis score in each group indicated that synovitis progressed with time but no significant differences were found in the same loading force groups at the same time point. a, b, c, and d: P < 0.01 (B). A representative micro-CT image showing synovial ectopic calcification is shown in which white arrows show the nodules around the injured knee (C). Quantification of ectopic nodules in each group showed higher number of calcified nodules in the 9N group compared to non-loaded knees and knees loaded with 6N force. There were no significant differences observed within the same loading groups. a, b and c P < 0.01 (D). L = lateral side, M = medial side, bars = 100 μm. Error bars represent 95% confidence interval. PBS = phosphate buffered saline, PRP = platelet-rich plasma.

We observed that non-loaded knees had normal synovium as before [25]. Both 6N and 9N loading resulted in some degree of synovitis (enlargement of synovial lining and increased density of cells in synovial stroma) at day 5 persisting to day 56 post-loading (Fig 5A). Quantification of synovitis score at day 5 and 56 post-injury indicated that synovitis was affected by time and loading force. At day 5 post-injury, 6N loaded + PBS treated group had a significant lower synovitis compared to 9N loaded + PBS treated group (Fig 5B, c: P < 0.01). However, the long term (56 day) results showed an increase in the severity of synovitis in 9N loaded + PBS treated group (Fig 5B, b: P < 0.01) compared to 6N loaded + PBS treated group (Fig 5B, a: P < 0.01). At each time point, the HA-derivative treated or PRP treated groups revealed no significant differences in synovitis compared to PBS groups, indicating that none of the treatment modality resolved synovitis (Fig 5B). We also observed the formation of calcified synovial nodules in the joint that were assessed by micro-CT (Fig 5C). These nodules were more pronounced in the 9N loading group compared to 6N loading group and non-loaded knees but we failed to observe any significant effect of HA-derivative and/or PRP treatments in resolving ectopic calcification (Fig 5D), even if the number of ectopic nodules decreased by 1/3 in comparison to PBS treated joints (in 9N group).

### Effect of treatment on bone parameters

**Fig 6:**
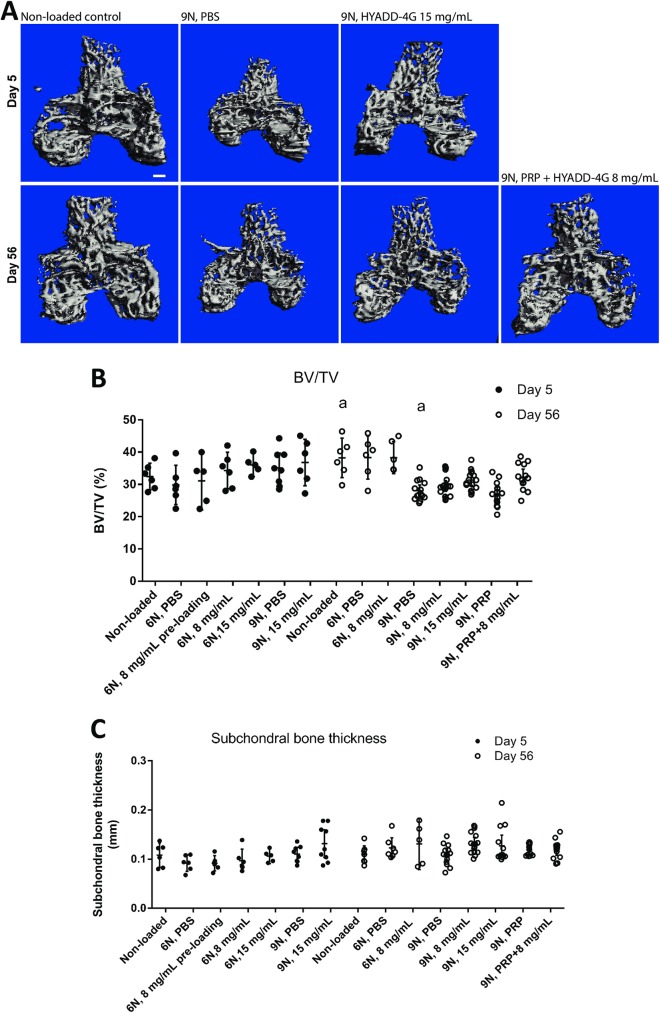
Micro-CT analysis. Micro-CT results showed no preventive effect of treatments on BV/TV at day 56 following loading. Trabecular bone was analyzed at the femoral epiphysis at day 5 (top row) and day 56 (bottom row). All images are presented as superior views (A). Quantification of femoral epiphysis trabecular bone volume (BV/TV) showed that there was no significant difference between treatment groups and the PBS at this time point (B). a: P = 0.02. Interestingly, at day 56 (9N group) receiving Hyadd-4G at 15 mg/mL and PRP + HYADD^®^ 4-G at 8 mg/mL group showed no statistical differences at the 56 days, non-loaded group. Quantification of subchondral bone thickness, showing no preventive effect of treatments on bone loss at 56 days after loading is presented (C). Error bars represent 95% confidence interval. PBS = phosphate buffered saline, PRP = platelet-rich plasma.

We observed some differences in bone volume fraction (BV/TV) between non-loaded and loaded knees, but the only significant difference was noted between non-loaded knees and 9N loaded knees at 56 days (Fig 6A and 6B). No appreciable differences were seen in subchondral bone plate thickness between loaded and non-loaded knees (Fig 6C). The subtle difference in BV/TV in loaded and non-loaded knees were mitigated in HA-derivative at 8 mg/mL + PRP group and in HA-derivative at 15 mg/mL group without reaching statistical significance. Furthermore, no significant differences in these groups were seen in comparison to non-loaded joints.

## Discussion

In this study, we used known short-term parameters of the compression model to determine whether there were effects of HA-derivative and/or PRP. In addition, we extended the time after injury to 8 weeks (56 days) to determine long-term consequences of the single injury and whether there were long-term effects of the treatments.

This study confirmed our previous findings in the compression model, which was largely characterized by the focal loss of proteoglycan at the site of cartilage injury on the posterior aspect of femoral condyle, chondrocyte apoptosis in this area, changes in the pattern of aggrecan expression, synovitis and ectopic calcification at early time points [24, 25]. We did not find pronounced progression in cartilage loss, cartilage fibrillation, and PTOA with 9N loading even over 56-day time period. This observation is in definite contrast with the DMM (destabilization of the medial meniscus) model of OA in which the medial meniscal tibial ligament is disrupted through a surgical procedure. In loading model, while chondrocytes in the injured area have a high degree of cell death, the cartilage remains intact (other than complete loss of Safranin-O staining in the non-calcified cartilage extending to <50% of the articular surface) even 8 weeks post-injury.

Several new studies have shown that acute chondrocyte death caused by in vitro impact injuries results from apoptosis [24, 34]. Lisignoli et al. [10] and Zhou et al. [35] have shown that HA can inhibit chondrocyte apoptosis via CD44 and CD54 activation. In our study, regardless of the injection time (e.g. pre-loading or post-loading), dosage of HA-derivative (e.g. 8 mg/mL or 15 mg/mL) ± PRP and loading force (e.g. 6N or 9N) did not reveal any obvious immediate preventive effect towards chondrocyte apoptosis emerging from tibial compression. This discrepancy could be attributed to the nature of the factors (experimental conditions) that triggered the apoptotic process. In our study, in vivo mechanical loading triggered apoptosis, while Fas ligand or IL-1β stimulated chondrocyte apoptosis in the aforementioned in vitro studies.

Our findings differ with other studies that have reported a beneficial effect of repeated injections of both HA and PRP in the rabbit joints after impact to patella and femoral condyle [36, 37]. In these studies, cell apoptosis was found to be significantly reduced after repeated (total 8 injections) intra-articular injections of 1 mg/kg HA at 30 days after knee impact, or at 10 days after a single post-loading injections of PRP. No other parameters were evaluated in these studies to detect the whole joint damage, and the differences in observations compared to our study could be related to the magnitude and frequency of compression applied during the impact loading, severity of joint damage or differences in animal model. In another study [38], presence of HA and chondroitin sulphate reduced the number of apoptosed chondrocytes in equine cartilage explants after single-impact loading. However, no changes in type II collagen or proteoglycan metabolism were noticed.

HA interacts with its surface receptors, CD44, on chondrocytes [10]. CD44-HA interactions link chondrocytes to their matrix and these interactions modulate cartilage metabolism [39]. Inhibition of CD44 expression causes substantial loss of aggrecan and promotes matrix remodeling [40]. Our previous study has shown a change in the pattern of CD44 expression in the injured cartilage [25] at early time-point after loading, with an increased expression a few days after impact. The data of PBS group in the current study are consistent with our previous findings [25] and no difference was evident between PBS, HA-derivative and PRP treated animals for CD44 expression on chondrocytes. Together with our observation, it suggests that the early events after impact loading do not seem to be modified by exogenous HA.

The role of ectopic ossification and osteophytes in OA development is not completely clear, although it is thought that they mechanically stabilize an osteoarthritic joint, thereby preventing structural progression [41]. Felson and co-workers [42] have shown that osteophytes are predictors for progression of OA, due to their strong association with joint malalignment. In a number of clinical investigations, it has been reported that osteophytes are strongly associated with OA joint pain and its progression [43]. In our study, histological and micro-CT evaluations revealed a reduction, though not statistically significant, in ectopic calcification/osteophyte formation at the 8-week time point after HA-derivative and PRP injections. Consequently, HA-derivative may relieve OA pain in part by reducing ectopic calcification and osteophyte formation. Additional preclinical and clinical studies will be required to test this hypothesis.

Christiansen and colleagues [5] have reported changes in knee joint following a one-time 12N loading as they observed a rapid loss of trabecular bone in injured knees at day 7 post-loading, which persisted until day 56. We also observed a loss of trabecular bone only at 56-day time point in the mice that underwent 9N loading. Since we used a lower loading force, our short-term (day 5) data did not show any significant changes in bone volume fraction and subchondral bone plate thickness. The effect of HA on bone formation/remodeling remains controversial. Sasaki and Watanabe [44] showed that high molecular weight HA was osteoinductive, while Cho et al. [45] demonstrated that HA failed to promote bone formation in distraction osteogenesis. Interestingly, we did not find any significant effect of HA-derivative alone or in combination with PRP on trabecular bone volume fraction. This lack of effect can be attributed to an increase weight bearing in the joints of the HA-derivative treated groups.

Numerous PRP studies have been performed in laboratory and clinical research in last two decades. Of these, some studies have reported that PRP increases chondrocyte viability and proliferation [46], and enhances the deposition of extracellular matrix proteins [47]. Other studies have reported PRP decreased type II collagen synthesis and stimulated the expression of catabolic (inflammatory) molecules [48]. One potential reason for these disparities could be attributed to the variations in the composition of PRP, including different protocols employed for the preparation and activation of PRP.

Although PRP has been used for over 40 years for treatment of acute and chronic injuries of bone and cartilage [49], only a few studies have shown its therapeutic effect in injury compared to HA [50, 51], thus leaving the therapeutic efficacy of PRP in OA treatment controversial. The combination of HA+PRP has been proposed as a therapy by Chen et al. [52] for tissue regeneration in OA. The therapeutic efficacy of the combined products was demonstrated in 3-dimensional arthritic neo-cartilage and in an OA model of ACL tear. The cartilaginous extracellular matrix was retrieved from inflammation-induced degradation by HA+PRP. The intra-articular injection of HA+PRP could strongly rescue the meniscus tear and cartilage breakdown and promote cartilage regeneration and inhibit inflammation. In our study, the combination of HA-derivative and PRP may indicate that the association can have synergistic effect only on the trabecular bone loss at longer term after knee injury.

This study has some limitations. There are no studies to directly compare the effect of PRP or HA with our study, so we used the literature from other study designs such as the in vitro effects of HA in IL-1β-treated human chondrocytes, in vitro equine impaction-induced apoptosis and in vivo loading induced apoptosis in rabbit joints after impact to patella and femoral condyle. It is likely that HA has effect on other functional parameters that are beyond the scope of present study. Therefore, our findings do not represent direct comparison with the other studies. Our study also lacks appropriate positive control, as there are currently no disease modifying OA drugs or the use of some treatments has been evaluated for functional outcomes such pain and gait pattern, which we did not undertake in our study. Furthermore, the analyses were performed on the femur side as this model results in changes that occur on the femur. It would have been ideal if our study could provide more specific information on focal subchondral or trabecular bone analyses given the focal nature of the joint injury, however, this was not possible, as we could not detect any focal changes in the bone on micro-CT. Lastly, the outcomes of our study were centered on joint structure, whereas clinical findings on the effectiveness of HA and PRP are often focused on patient-reported outcome measures (symptoms) [53] or biomarker assessment [54]. To quantify these parameters in rodent studies, other outcome measures will be used such as gait analysis, pain assessment and serum and/or synovial biomarkers. On the flip side, patient reported outcome measures ascertain whether patients feel better with HA or PRP treatment, whereas in animal studies we can measure actual structural changes.

### Conclusions

In this study, contrary to our hypothesis, there was no protective effect of HA-derivative (HYADD^®^ 4-G) at two different concentrations (8 mg/mL and 15 mg/mL) at two different loadings (6N and 9N) and at two different time points (5 days and 56 days) on these parameters: cartilage proteoglycan loss, chondrocyte apoptosis, expression pattern of aggrecan, COMP, and CD44, synovitis, ectopic calcification, subchondral bone plate thickness. Bone volume trabecular fraction was found to be significantly different between loaded (9N group) and non-loaded joints. The repeated injections of HA-derivative at 15 mg/mL and HA-derivative at 8 mg/mL+PRP, could reduce but not significantly improve the bone changes. Additionally, the injections of only PRP conferred no therapeutic efficacy, and the results were not significantly different from PBS. Other potential treatment options need to be investigated to reduce the deleterious immediate and long-term effects of traumatic injury to the knee joint.

## Supporting information

S1 Fig Non-immune controls for TUNEL assay and CD44 staining. **A.** Non-immune control for TUNEL assay. **B**. Non-immune control for CD44 staining. The area indicated by white dotted line boxes is shown at higher magnification on the right. The dotted lines indicate the edges of the cartilages. Bars = 100 μm.
(TIF)

S2 Fig Split channels of CD44 staining. **A**. Images with split channels from Fig 4C. **B**. Images with split channels from Fig 4D. Red = aggrecan, green = CD44, blue = DAPI. Bars = 100 μm.
(TIF)

## References

[1] Brown TD, Johnston RC, Saltzman CL, Marsh JL, Buckwalter JA. Posttraumatic osteoarthritis: a first estimate of incidence, prevalence, and burden of disease. J Orthop Trauma. 2006;20(10):739–44. Epub 2006/11/16. doi: 10.1097/01.bot.0000246468.80635.ef 17106388 10.1097/01.bot.0000246468.80635.ef

[2] Joosten LA, Smeets RL, Koenders MI, van den Bersselaar LA, Helsen MM, Oppers-Walgreen B, et al Interleukin-18 promotes joint inflammation and induces interleukin-1-driven cartilage destruction. Am J Pathol. 2004;165(3):959–67. Epub 2004/08/28. PubMed Central PMCID: PMC1618596. doi: 10.1016/S0002-9440(10)63357-3 15331419 10.1016/S0002-9440(10)63357-3 PMC1618596

[3] Glasson SS, Blanchet TJ, Morris EA. The surgical destabilization of the medial meniscus (DMM) model of osteoarthritis in the 129/SvEv mouse. Osteoarthritis Cartilage. 2007;15(9):1061–9. Epub 2007/05/02. doi: 10.1016/j.joca.2007.03.006 17470400 10.1016/j.joca.2007.03.006

[4] Kamekura S, Hoshi K, Shimoaka T, Chung U, Chikuda H, Yamada T, et al Osteoarthritis development in novel experimental mouse models induced by knee joint instability. Osteoarthritis Cartilage. 2005;13(7):632–41. doi: 10.1016/j.joca.2005.03.004 15896985 10.1016/j.joca.2005.03.004

[5] Christiansen BA, Anderson MJ, Lee CA, Williams JC, Yik JH, Haudenschild DR. Musculoskeletal changes following non-invasive knee injury using a novel mouse model of post-traumatic osteoarthritis. Osteoarthritis Cartilage. 2012;20(7):773–82. Epub 2012/04/26. doi: 10.1016/j.joca.2012.04.014 22531459 10.1016/j.joca.2012.04.014

[6] Poulet B, Hamilton RW, Shefelbine S, Pitsillides AA. Characterizing a novel and adjustable noninvasive murine joint loading model. Arthritis Rheum. 2011;63(1):137–47. Epub 2010/10/01. doi: 10.1002/art.27765 20882669 10.1002/art.27765

[7] Balazs EA, Denlinger JL. Viscosupplementation: a new concept in the treatment of osteoarthritis. J Rheumatol Suppl. 1993;39:3–9. 8410881

[8] Gigante A, Callegari L. The role of intra-articular hyaluronan (Sinovial) in the treatment of osteoarthritis. Rheumatol Int. 2011;31(4):427–44. doi: 10.1007/s00296-010-1660-6 21113807 10.1007/s00296-010-1660-6

[9] Masuko K, Murata M, Yudoh K, Kato T, Nakamura H. Anti-inflammatory effects of hyaluronan in arthritis therapy: Not just for viscosity. Int J Gen Med. 2009;2:77–81. PubMed Central PMCID: PMC2840553. 20360891 10.2147/ijgm.s5495 PMC2840553

[10] Lisignoli G, Grassi F, Zini N, Toneguzzi S, Piacentini A, Guidolin D, et al Anti-Fas-induced apoptosis in chondrocytes reduced by hyaluronan: evidence for CD44 and CD54 (intercellular adhesion molecule 1) invovement. Arthritis Rheum. 2001;44(8):1800–7. doi: 10.1002/1529-0131(200108)44:8<1800::AID-ART317>3.0.CO;2-1 11508432 10.1002/1529-0131(200108)44:8<1800::AID-ART317>3.0.CO;2-1

[11] Petrella RJ, Petrella M. A prospective, randomized, double-blind, placebo controlled study to evaluate the efficacy of intraarticular hyaluronic acid for osteoarthritis of the knee. J Rheumatol. 2006;33(5):951–6. 16652426

[12] Mainil-Varlet P, Schiavinato A, Ganster MM. Efficacy Evaluation of a New Hyaluronan Derivative HYADD 4-G to Maintain Cartilage Integrity in a Rabbit Model of Osteoarthritis. Cartilage. 2013;4(1):28–41. PubMed Central PMCID: PMC3583149. doi: 10.1177/1947603512455193 23550192 10.1177/1947603512455193 PMC3583149

[13] Heybeli N, Doral MN, Atay OA, Leblebicioglu G, Uzumcugil A. [Intra-articular sodium hyaluronate injections after arthroscopic debridement for osteoarthritis of the knee: a prospective, randomized, controlled study]. Acta Orthop Traumatol Turc. 2008;42(4):221–7. 19060514 10.3944/aott.2008.221

[14] Porcellini G, Merolla G, Giordan N, Paladini P, Burini A, Cesari E, et al Intra-articular glenohumeral injections of HYADD(R)4-G for the treatment of painful shoulder osteoarthritis: a prospective multicenter, open-label trial. Joints. 2015;3(3):116–21. PubMed Central PMCID: PMC4732777. doi: 10.11138/jts/2015.3.3.116 26889467 10.11138/jts/2015.3.3.116 PMC4732777

[15] Bonnevie ED, Galesso D, Secchieri C, Cohen I, Bonassar LJ. Elastoviscous Transitions of Articular Cartilage Reveal a Mechanism of Synergy between Lubricin and Hyaluronic Acid. PLoS One. 2015;10(11):e0143415 PubMed Central PMCID: PMCPMC4658013. doi: 10.1371/journal.pone.0143415 26599797 10.1371/journal.pone.0143415 PMC4658013

[16] Schmidt MB, Chen EH, Lynch SE. A review of the effects of insulin-like growth factor and platelet derived growth factor on in vivo cartilage healing and repair. Osteoarthritis Cartilage. 2006;14(5):403–12. doi: 10.1016/j.joca.2005.10.011 16413799 10.1016/j.joca.2005.10.011

[17] Li TF, O'Keefe RJ, Chen D. TGF-beta signaling in chondrocytes. Front Biosci. 2005;10:681–8. PubMed Central PMCID: PMC2647990. 15569609 10.2741/1563 PMC2647990

[18] Khoshbin A, Leroux T, Wasserstein D, Marks P, Theodoropoulos J, Ogilvie-Harris D, et al The efficacy of platelet-rich plasma in the treatment of symptomatic knee osteoarthritis: a systematic review with quantitative synthesis. Arthroscopy. 2013;29(12):2037–48. doi: 10.1016/j.arthro.2013.09.006 24286802 10.1016/j.arthro.2013.09.006

[19] Muramatsu Y, Sasho T, Saito M, Yamaguchi S, Akagi R, Mukoyama S, et al Preventive effects of hyaluronan from deterioration of gait parameters in surgically induced mice osteoarthritic knee model. Osteoarthritis Cartilage. 2014;22(6):831–5. doi: 10.1016/j.joca.2014.03.016 24704496 10.1016/j.joca.2014.03.016

[20] Elmorsy S, Funakoshi T, Sasazawa F, Todoh M, Tadano S, Iwasaki N. Chondroprotective effects of high-molecular-weight cross-linked hyaluronic acid in a rabbit knee osteoarthritis model. Osteoarthritis Cartilage. 2014;22(1):121–7. doi: 10.1016/j.joca.2013.10.005 24185110 10.1016/j.joca.2013.10.005

[21] Kazemi D, Fakhrjou A. Leukocyte and Platelet Rich Plasma (L-PRP) Versus Leukocyte and Platelet Rich Fibrin (L-PRF) For Articular Cartilage Repair of the Knee: A Comparative Evaluation in an Animal Model. Iran Red Crescent Med J. 2015;17(10):e19594 PubMed Central PMCID: PMC4640060. doi: 10.5812/ircmj.19594 26568857 10.5812/ircmj.19594 PMC4640060

[22] Takebe K, Rai MF, Schmidt EJ, Sandell LJ. The chemokine receptor CCR5 plays a role in post-traumatic cartilage loss in mice, but does not affect synovium and bone. Osteoarthritis Cartilage. 2015;23(3):454–61. PubMed Central PMCID: PMC4341917. doi: 10.1016/j.joca.2014.12.002 25498590 10.1016/j.joca.2014.12.002 PMC4341917

[23] Sonmez TT, Vinogradov A, Zor F, Kweider N, Lippross S, Liehn EA, et al The effect of platelet rich plasma on angiogenesis in ischemic flaps in VEGFR2-luc mice. Biomaterials. 2013;34(11):2674–82. doi: 10.1016/j.biomaterials.2013.01.016 23352038 10.1016/j.biomaterials.2013.01.016

[24] Wu P, Holguin N, Silva MJ, Fu M, Liao W, Sandell LJ. Early response of mouse joint tissue to noninvasive knee injury suggests treatment targets. Arthritis Rheumatol. 2014;66(5):1256–65. doi: 10.1002/art.38375 24470303 10.1002/art.38375 PMC4310559

[25] Duan X, Rai MF, Holguin N, Silva MJ, Patra D, Liao W, et al Early changes in the knee of healer and non-healer mice following non-invasive mechanical injury. J Orthop Res. 2017;35(3):524–536. doi: 10.1002/jor.23413 27591401 10.1002/jor.23413 PMC5718184

[26] Diekman BO, Wu CL, Louer CR, Furman BD, Huebner JL, Kraus VB, et al Intra-articular delivery of purified mesenchymal stem cells from C57BL/6 or MRL/MpJ superhealer mice prevents posttraumatic arthritis. Cell Transplant. 2013;22(8):1395–408. PubMed Central PMCID: PMC3891895. doi: 10.3727/096368912X653264 22889498 10.3727/096368912X653264 PMC3891895

[27] van der Kraan PM, Vitters EL, van Beuningen HM, van de Putte LB, van den Berg WB. Degenerative knee joint lesions in mice after a single intra-articular collagenase injection. A new model of osteoarthritis. J Exp Pathol (Oxford). 1990;71(1):19–31. PubMed Central PMCID: PMC1998679. 2155638 PMC1998679

[28] Hashimoto S, Rai MF, Janiszak KL, Cheverud JM, Sandell LJ. Cartilage and bone changes during development of post-traumatic osteoarthritis in selected LGXSM recombinant inbred mice. Osteoarthritis Cartilage. 2012;20(6):562–71. PubMed Central PMCID: PMC3353722. doi: 10.1016/j.joca.2012.01.022 22361237 10.1016/j.joca.2012.01.022 PMC3353722

[29] Hildebrand T, Rüegsegger P. A new method for the model-independent assessment of thickness in three-dimensional images. Journal of Microscopy. 1997;185(1):67–75.

[30] Glasson SS, Chambers MG, Van Den Berg WB, Little CB. The OARSI histopathology initiative—recommendations for histological assessments of osteoarthritis in the mouse. Osteoarthritis Cartilage. 2010;18 Suppl 3:S17–23. 10.1016/j.joca.2010.05.025 20864019

[31] Lewis JS, Hembree WC, Furman BD, Tippets L, Cattel D, Huebner JL, et al Acute joint pathology and synovial inflammation is associated with increased intra-articular fracture severity in the mouse knee. Osteoarthritis Cartilage. 2011;19(7):864–73. Epub 2011/05/31. PubMed Central PMCID: PMC3312469. doi: 10.1016/j.joca.2011.04.011 21619936 10.1016/j.joca.2011.04.011 PMC3312469

[32] Farnum CE, Turgai J, Wilsman NJ. Visualization of living terminal hypertrophic chondrocytes of growth plate cartilage in situ by differential interference contrast microscopy and time-lapse cinematography. J Orthop Res. 1990;8:750–63. doi: 10.1002/jor.1100080517 2201757 10.1002/jor.1100080517

[33] D'Lima DD, Hashimoto S, Chen PC, Colwell CW Jr., Lotz MK. Human chondrocyte apoptosis in response to mechanical injury. Osteoarthritis Cartilage. 2001;9(8):712–9. doi: 10.1053/joca.2001.0468 11795990 10.1053/joca.2001.0468

[34] Goodwin W, McCabe D, Sauter E, Reese E, Walter M, Buckwalter JA, et al Rotenone prevents impact-induced chondrocyte death. J Orthop Res. 2010;28(8):1057–63. PubMed Central PMCID: PMC3678274. doi: 10.1002/jor.21091 20108345 10.1002/jor.21091 PMC3678274

[35] Zhou PH, Liu SQ, Peng H. The effect of hyaluronic acid on IL-1beta-induced chondrocyte apoptosis in a rat model of osteoarthritis. J Orthop Res. 2008;26(12):1643–8. doi: 10.1002/jor.20683 18524010 10.1002/jor.20683

[36] Barreto RB, Sadigursky D, de Rezende MU, Hernandez AJ. Effect of hyaluronic acid on chondrocyte apoptosis. Acta Ortop Bras. 2015;23(2):90–3. PubMed Central PMCID: PMC4813412. doi: 10.1590/1413-785220152302144341 27069407 10.1590/1413-785220152302144341 PMC4813412

[37] de-Rezende MU, Silva RBBd, Bassit ACF, Tatsui NH, Sadigursky D, Neto RB. Effect of platelet-rich plasma on impact-induced chondrocyte apoptosis. Acta Ortopédica Brasileira. 2011;19(2):201–5.

[38] Henson FM, Getgood AM, Caborn DM, McIlwraith CW, Rushton N. Effect of a solution of hyaluronic acid-chondroitin sulfate-N-acetyl glucosamine on the repair response of cartilage to single-impact load damage. Am J Vet Res. 2012;73(2):306–12. doi: 10.2460/ajvr.73.2.306 22280395 10.2460/ajvr.73.2.306

[39] Knudson CB, Knudson W. Hyaluronan and CD44: modulators of chondrocyte metabolism. Clin Orthop Relat Res. 2004;(427 Suppl):S152–62. 15480059

[40] Chow G, Knudson CB, Homandberg G, Knudson W. Increased expression of CD44 in bovine articular chondrocytes by catabolic cellular mediators. J Biol Chem. 1995;270(46):27734–41. 7499241 10.1074/jbc.270.46.27734

[41] Guyton GP, Brand RA. Apparent spontaneous joint restoration in hip osteoarthritis. Clin Orthop Relat Res. 2002;(404):302–7. 12439273 10.1097/00003086-200211000-00045

[42] Felson DT, Gale DR, Elon Gale M, Niu J, Hunter DJ, Goggins J, et al Osteophytes and progression of knee osteoarthritis. Rheumatology (Oxford). 2005;44(1):100–4. 15381791 10.1093/rheumatology/keh411

[43] Lanyon P, O'Reilly S, Jones A, Doherty M. Radiographic assessment of symptomatic knee osteoarthritis in the community: definitions and normal joint space. Ann Rheum Dis. 1998;57(10):595–601. PubMed Central PMCID: PMC1752476. 9893570 10.1136/ard.57.10.595 PMC1752476

[44] Sasaki T, Watanabe C. Stimulation of osteoinduction in bone wound healing by high-molecular hyaluronic acid. Bone. 1995;16(1):9–15. 7742090 10.1016/s8756-3282(94)00001-8

[45] Cho BC, Park JW, Baik BS, Kwon IC, Kim IS. The role of hyaluronic acid, chitosan, and calcium sulfate and their combined effect on early bony consolidation in distraction osteogenesis of a canine model. J Craniofac Surg. 2002;13(6):783–93. 12457095 10.1097/00001665-200211000-00014

[46] Park SI, Lee HR, Kim S, Ahn MW, Do SH. Time-sequential modulation in expression of growth factors from platelet-rich plasma (PRP) on the chondrocyte cultures. Mol Cell Biochem. 2012;361(1–2):9–17. doi: 10.1007/s11010-011-1081-1 21956670 10.1007/s11010-011-1081-1

[47] Kruger JP, Hondke S, Endres M, Pruss A, Siclari A, Kaps C. Human platelet-rich plasma stimulates migration and chondrogenic differentiation of human subchondral progenitor cells. J Orthop Res. 2012;30(6):845–52. doi: 10.1002/jor.22005 22058056 10.1002/jor.22005

[48] Lee HR, Shon OJ, Park SI, Kim HJ, Kim S, Ahn MW, et al Platelet-Rich Plasma Increases the Levels of Catabolic Molecules and Cellular Dedifferentiation in the Meniscus of a Rabbit Model. Int J Mol Sci. 2016;17(1). PubMed Central PMCID: PMC4730361. 10.3390/ijms17010120 PMC4730361 26784189

[49] Andia I, Maffulli N. Platelet-rich plasma for managing pain and inflammation in osteoarthritis. Nat Rev Rheumatol. 2013;9(12):721–30. doi: 10.1038/nrrheum.2013.141 24080861 10.1038/nrrheum.2013.141

[50] Spakova T, Rosocha J, Lacko M, Harvanova D, Gharaibeh A. Treatment of knee joint osteoarthritis with autologous platelet-rich plasma in comparison with hyaluronic acid. Am J Phys Med Rehabil. 2012;91(5):411–7. doi: 10.1097/PHM.0b013e3182aab72 22513879 10.1097/PHM.0b013e3182aab72

[51] Sanchez M, Fiz N, Azofra J, Usabiaga J, Aduriz Recalde E, Garcia Gutierrez A, et al A randomized clinical trial evaluating plasma rich in growth factors (PRGF-Endoret) versus hyaluronic acid in the short-term treatment of symptomatic knee osteoarthritis. Arthroscopy. 2012;28(8):1070–8. doi: 10.1016/j.arthro.2012.05.011 22840987 10.1016/j.arthro.2012.05.011

[52] Chen WH, Lo WC, Hsu WC, Wei HJ, Liu HY, Lee CH, et al Synergistic anabolic actions of hyaluronic acid and platelet-rich plasma on cartilage regeneration in osteoarthritis therapy. Biomaterials. 2014;35(36):9599–607. doi: 10.1016/j.biomaterials.2014.07.058 25176059 10.1016/j.biomaterials.2014.07.058

[53] Raeissadat SA, Rayegani SM, Hassanabadi H, Fathi M, Ghorbani E, Babaee M, et al Knee Osteoarthritis Injection Choices: Platelet- Rich Plasma (PRP) Versus Hyaluronic Acid (A one-year randomized clinical trial). Clin Med Insights Arthritis Musculoskelet Disord. 2015;8:1–8. PubMed Central PMCID: PMCPMC4287055. doi: 10.4137/CMAMD.S17894 25624776 10.4137/CMAMD.S17894 PMC4287055

[54] Wasterlain AS, Braun HJ, Harris AH, Kim HJ, Dragoo JL. The systemic effects of platelet-rich plasma injection. Am J Sports Med. 2013;41(1):186–93. doi: 10.1177/0363546512466383 23211708 10.1177/0363546512466383

