# Supplementary figures and images for: Therapeutic efficacy of intra-articular hyaluronan derivative and platelet-rich plasma in mice following axial tibial loading
Source: PLoS One. 2017 Apr 13;12(4):e0175682. doi: 10.1371/journal.pone.0175682 (PMC5391072; doi:10.1371/journal.pone.0175682)

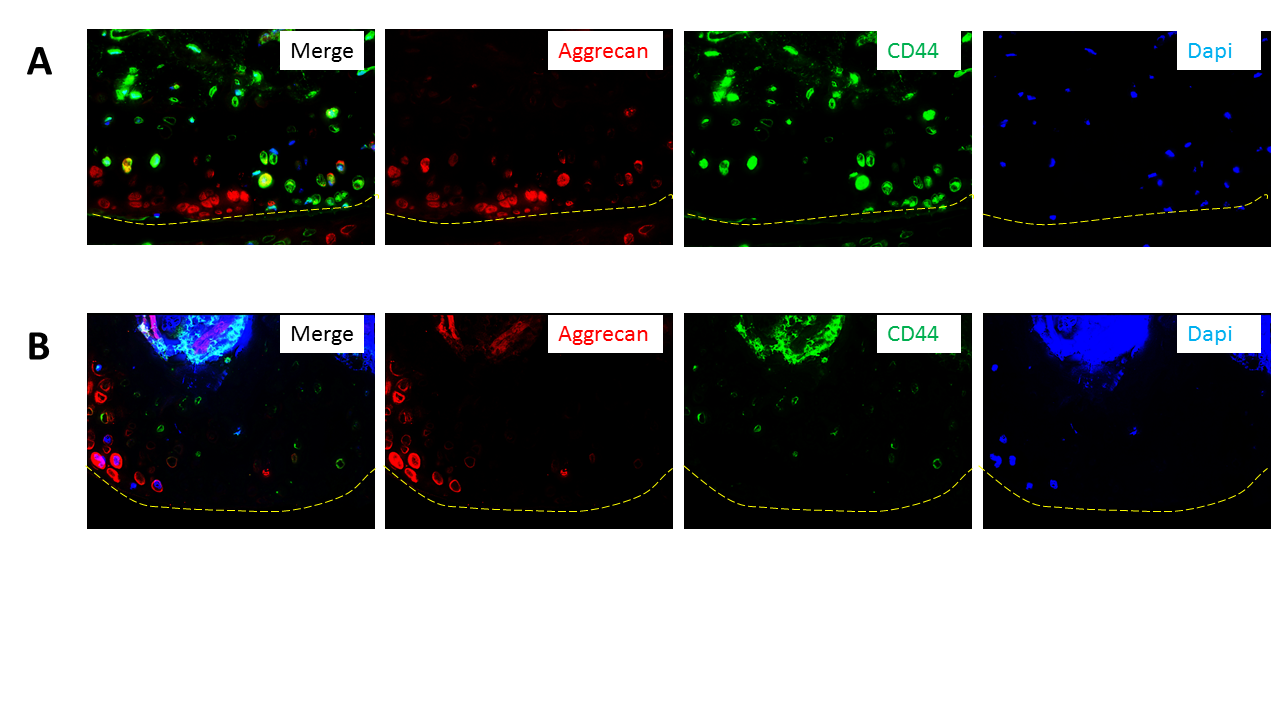

Supplement: S1 Fig — A. Non-immune control for TUNEL assay. B. Non-immune control for CD44 staining. The area indicated by white dotted line boxes is shown at higher magnification on the right. The dotted lines indicate the edges of the cartilages. Bars = 100 μm. (TIF) [file pone.0175682.s001.TIF]

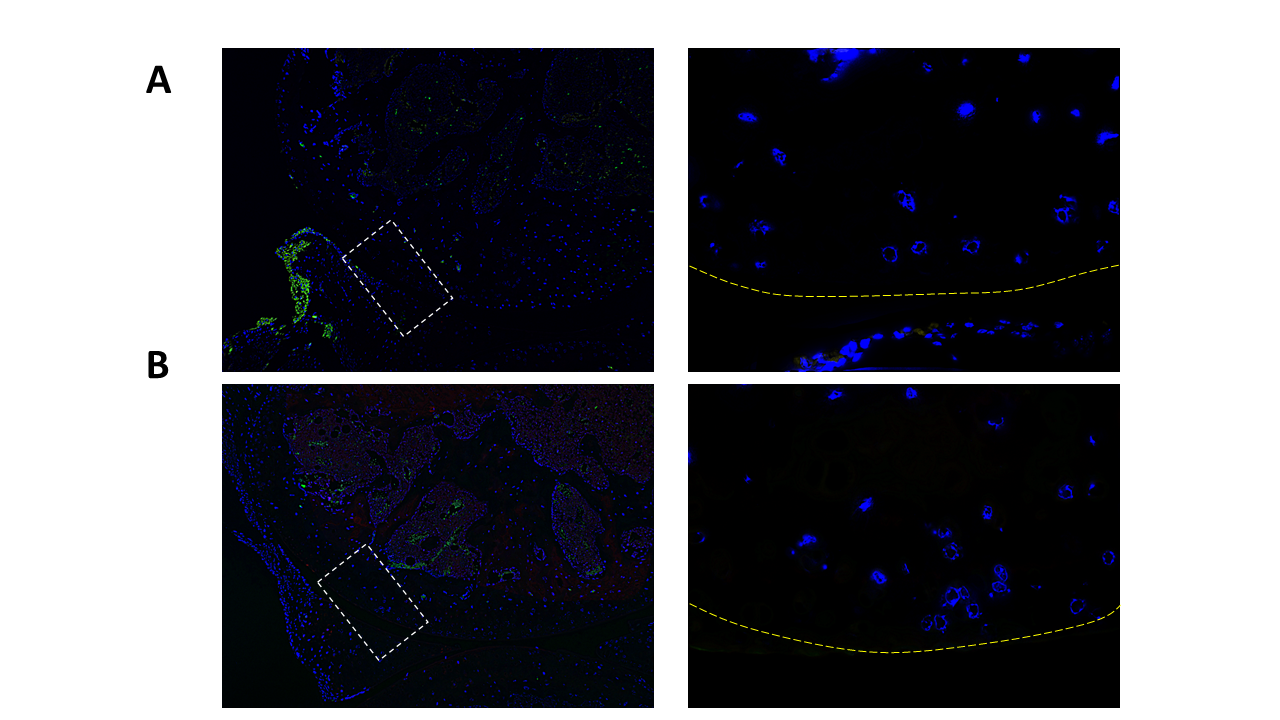

Supplement: S2 Fig — A. Images with split channels from Fig 4C. B. Images with split channels from Fig 4D. Red = aggrecan, green = CD44, blue = DAPI. Bars = 100 μm. (TIF) [file pone.0175682.s002.TIF]
